# Salivating for Knowledge: Potential Pharmacological Agents in Tick Saliva

**DOI:** 10.1371/journal.pmed.0050043

**Published:** 2008-02-12

**Authors:** Joppe W. R Hovius, Marcel Levi, Erol Fikrig

## Abstract

Joppe Hovius and colleagues review anticoagulant and immunosuppressive proteins present in tick saliva, and discuss how immunologically targeting such molecules could prevent transmission of tick-borne pathogens.

The incidence of tick-borne diseases has drastically increased over the past few years [1,2], resulting in a marked increase in research on tick–host–pathogen interactions. As a result, the knowledge on molecules present in tick saliva and their function has significantly expanded [3,4]. Ticks are obligate hematophagous ectoparasites, and hundreds of tick species are distributed worldwide. While taking a blood meal, ticks are attached to their host for several days and introduce saliva into the host skin. Like saliva from other hematophagous animals, such as mosquitoes, flies, leeches, and nematode species, tick saliva contains a wide range of physiologically active molecules that are crucial for attachment to the host or for the transmission of pathogens [5], and that interact with host processes, including coagulation and fibrinolysis, immunity and inflammation, and angiogenesis [3,6,7]. In this article, we discuss molecules in tick saliva that have been intensively studied in vitro or in animal models for human diseases, and that, due to their specificity, are potential future anticoagulant or immunosuppressive agents. We also discuss how immunologically targeting specific tick salivary proteins could prevent the transmission of tick-borne pathogens from the tick to the host.

## Anticoagulants

**Figure 1:**
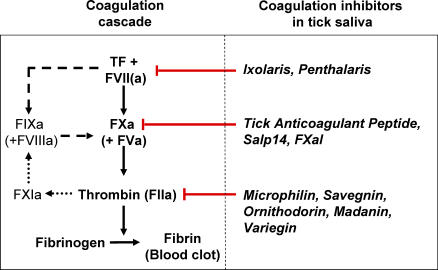
Schematic Overview of the Coagulation Cascade The two major amplification loops in the coagulation cascade are depicted. The first amplification loop consists of TF-FVIIa-mediated factor IX (FIX) activation, which leads to the generation of more FXa. A second amplification loop is formed by the activation of factor XI (FXIa) by thrombin, which results in more activated FIX (FIXa), and, subsequently, additional FXa generation. The right panel indicates how selected tick proteins exert their anticoagulant effect. FIIa, activated factor II; FVa, activated factor V; FVIIIa, activated factor VIII.

The hemostatic response enables mammals to control blood loss during vascular injury. Platelets adhere to macromolecules in exposed subendothelial tissue and aggregate to form a hemostatic plug, while local activation of plasma coagulation factors leads to generation of a fibrin clot that reinforces the platelet aggregate. The coagulation cascade starts when exposed subendothelial tissue factor (TF) binds to activated factor VII (FVIIa). This complex activates factor X (forming FXa), which mediates the formation of minute amounts of thrombin that activate other coagulation proteases and additional platelets. Subsequently, by means of two amplification loops (Figure 1), more thrombin is generated, which leads to fibrinogen-to-fibrin conversion and fibrin deposition [8].

Tick feeding is hampered by the hemostatic response of the host. Therefore tick saliva contains an extensive selection of molecules that counteract coagulation, enhance fibrinolysis, and inhibit platelet aggregation [7]. Traditional anticoagulant agents such as unfractionated heparin and vitamin K antagonists (e.g., warfarin) have a narrow therapeutic index, requiring frequent monitoring and dose adjustments [7]. Tick saliva presents a possible source of novel, and ideally more easily used, anticoagulant agents (Figure 1) [7].

**Five Key Papers in the Field**

**Hepburn et al., 2007** [40] After identification of a specific activated C5 inhibitor, OMCI, the authors showed how this protein can be used in an experimental animal model for myasthenia gravis.

**Paveglio et al., 2007** [50] Showed that a T cell inhibitor from tick saliva, Salp15, is able to prevent the development of pathological features in an animal model for atopic asthma.

**Labuda et al., 2006** [55] Showed that an anti-tick vaccine, directed against the 64TRP cement protein in tick saliva, prevented lethal infection of mice with the tick-borne encephalitis virus, indicating that anti-tick vaccines could be used to combat tick-borne pathogens.

**Ramamoorthi et al., 2005** [5] Showed that B. burgdorferi, the causative agent of Lyme disease, uses a protein in tick saliva, Salp15, to establish an infection in the mammalian host, underscoring the complex tick–host–pathogen interactions that are involved in the development of Lyme disease.

**Waxman et al., 1990** [9] Identified the first highly specific activated factor X inhibitor in tick saliva, TAP. This research has been the inspiration for numerous researchers working in the field of coagulation.

### Factor Xa inhibitors.

Saliva from the soft tick Ornithodoros moubata contains a serine protease inhibitor of FXa—tick anticoagulant peptide (TAP). TAP is a tight-binding specific FXa inhibitor that inhibits clotting of human plasma ex vivo [9]. The inhibitory characteristics and the high selectivity of recombinant forms of TAP (rTAP) for FXa are due to the interaction of rTAP with the active site as well as with regions remote from the active site pocket of FXa [10]. rTAP has been tested in a variety of animal models for both venous and arterial thrombosis [11–13]. A recent study showed that rTAP, when fused to a single-chain antibody specifically targeting activated platelets (through binding to the platelet receptor GPIIb/IIIa), had highly effective antithrombotic properties in comparison to enoxaparin in a murine carotid artery thrombosis model. In addition, in contrast to conventional anticoagulants tested, the TAP–antibody fusion protein did not prolong bleeding time [14]. Future research should reveal whether this or similar approaches are equally effective and safe in humans. Other FXa inhibitors characterized in tick saliva are shown in Table 1 [15,16].

**Table 1:**
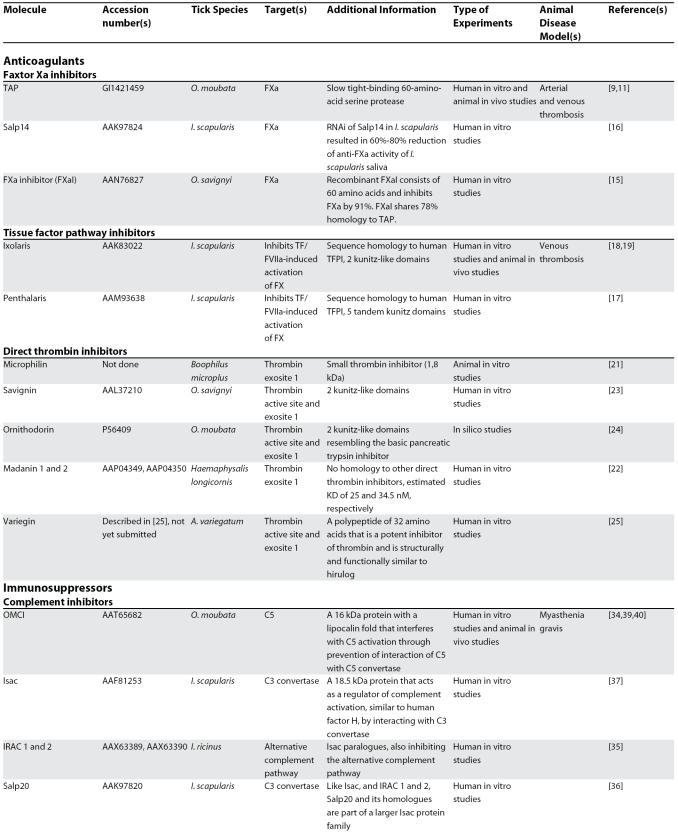
Anticoagulants and Immunosuppressors in Tick Saliva

**Table 1:**
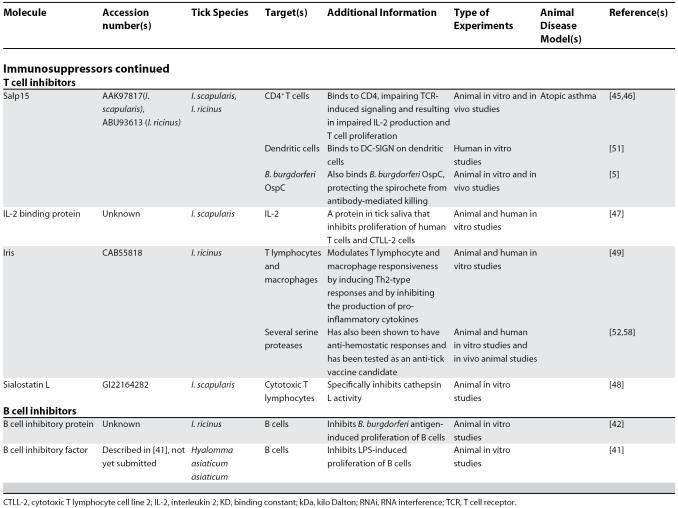
Continued.

### Tissue factor pathway inhibitors.

In view of the central role of TF in the initiation of coagulation in both physiological and pathological states, targeting TF may be an effective antithrombotic strategy. Tick saliva contains several TF pathway inhibitors (TFPIs) (Table 1) [7,17]. Recently, Ixolaris was identified in saliva from the deer tick Ixodes scapularis [17]. Ixolaris has two kunitz-like domains, a type of domain conserved in a wide family of serine protease inhibitors, and sequence homology to human TFPI [18]. In a rat model for venous thrombosis, administration of recombinant Ixolaris resulted in effective antithrombotic activity, without hemorrhage or bleeding [19]. Because of its fast and tight binding to FXa, giving rapid-acting, selective, and long-lasting effects, and the encouraging results in vivo, Ixolaris could serve as a template for potential new anticoagulant agents targeting the TF pathway.

### Direct thrombin inhibitors.

In comparison with heparin (derivatives), which act via antithrombin, direct thrombin inhibitors more effectively inhibit clot-bound thrombin, which is likely to result in a stronger antithrombotic effect [20]. Several specific direct thrombin inhibitors have been characterized in tick saliva (Table 1) [7,21–24], but most have not yet been tested in vivo. Recently a new direct thrombin inhibitor, variegin [25], was characterized from the tropical bont tick, Amblyomma variegatum, and shown to be structurally similar to, but much more potent than, hirulog, a 20-amino-acid synthetic thrombin inhibitor based on the natural leech peptide hirudin. Hirulog belongs to a class of drugs that have been approved for treatment of patients with acute coronary syndromes who are undergoing percutaneous coronary intervention [26].

## Immunosuppressors

Cellular innate immune responses, depending on invariant receptors such as the Toll-like receptors, are one of the first lines of defence against invading microbes. Another important innate defence system is the complement cascade. Activation of the complement system leads to opsonization of an invading pathogen as well as formation of the membrane attack complex that can lyse invading bacteria. The more specific adaptive immune response, which responds against pathogens that bypass the innate immune response, is triggered when activated antigen-presenting cells migrate to lymphoid tissue. In lymph nodes, antigen-presenting cells present processed antigen to T cells, which, upon activation, play a central role in cellular immune responses at the site of infection, or assist in the activation of B cells for the generation of an antigen-specific humoral response.

Ticks acquire a blood meal over a period of days, allowing the host sufficient time to generate anti-tick immune responses. The tick, in turn, has developed mechanisms to protect itself against host inflammation and immune responses [4]. In light of the central role of the complement cascade and T and B cells in many human diseases, we focus on specific tick salivary molecules that target these responses.

### Complement inhibitors.

The complement system is involved in the pathogenesis of many autoimmune diseases, including rheumatoid arthritis, systemic lupus erythematosus, and multiple sclerosis, and also in ischemia-reperfusion injury as observed in acute myocardial infarction or ischemic stroke [27–29]. Inhibitors of the complement cascade are therefore of potential clinical interest. Many agents inhibit complement factor 3 (C3) convertase early in the complement cascade, but this inhibition can result in immunosuppression, impairment of opsonization, or immune complex deposition. Novel complement inhibitors should therefore preferably inhibit the complement cascade downstream of complement factor 5 (C5), allowing the upstream cascade to proceed physiologically. Early randomized controlled clinical trials studying the effect of an antibody targeting C5 in acute myocardial infarction showed promising results [30,31], although a more recent randomized controlled trial showed no beneficial effect on all-cause mortality of a C5-antibody compared to placebo [32]. A similar antibody was shown to be effective in the treatment of autoimmune diseases [33].

Tick saliva contains many molecules that specifically inhibit complement activation (Table 1) [34–38]. A promising tick complement inhibitor is the C5 activation inhibitor from the soft tick O. moubata, OMCI [34,39]. OMCI inhibits C5 activation by interfering with C5 convertase [39], and has been shown to inhibit human complement hemolytic activity and the development of pathological features in a rodent model for autoimmune myasthenia gravis [40].

### B cell inhibitors.

The I. ricinus B cell inhibitory protein (BIP) is one of the tick salivary proteins that suppress proliferation of murine B cells (Table 1) [41,42]. Suppression of B cell responses benefits the tick by inhibiting specific anti-tick antibody responses that could lead to rejection by the host. In addition, B cells are unable to respond adequately to Borrelia burgdorferi antigens in the presence of BIP, suggesting that B. burgdorferi might also benefit from BIP-mediated B cell suppression. Specific inhibition of B cells has been shown to be effective in clinical studies of lymphoproliferative disorders and autoimmune diseases, such as rheumatoid arthritis and multiple sclerosis [43,44]. In order to serve as a template for novel drugs specifically targeting B cells, tick B cell inhibitors need further characterization.

### T cell inhibitors.

The I. scapularis 15 kDa salivary protein, Salp15, is an example of a feeding-induced protein that inhibits the activation of T cells (Table 1) [45–49]. Salp15 specifically binds to the CD4 molecule on CD4^+^ T (helper) cells, which results in inhibition of T cell receptor–mediated signaling, leading to reduced interleukin-2 production and impaired T cell proliferation [46]. In an experimental mouse model of allergic airway disease, Salp15 prevented the development of atopic asthma [50], suggesting that Salp15 might be used to modulate atopic disease as well as T cell–driven autoimmune diseases. We have shown that Salp15 also inhibits inflammatory cytokine production by human monocyte-derived dendritic cells by interacting with the C-type lectin receptor DC-SIGN [51], indicating that Salp15 has the potential to modulate human adaptive immune responses. Iris, an immunosuppressive protein from I. ricinus, has been shown to modulate T cell responses through inhibition of interferon- and to inhibit interleukin-6 and tumor necrosis factor-a production by human macrophages [49]. In addition, Iris also has been shown to have anti-hemostatic effects by inhibiting several serine proteases involved in the coagulation cascade and fibrinolysis [52].

## New Strategies to Prevent Tick-Borne Diseases

Understanding the importance of specific tick salivary proteins for attachment to the host and for transmission of pathogens may permit us to develop new strategies (e.g., anti-tick vaccines) for preventing tick-borne diseases. The idea of a tick-antigen-based vaccine is supported by the observation that repeated exposure of certain animals to tick bites results in an inability of ticks to successfully take a blood meal [45]. These animals, as well as humans who develop hypersensitivity after repeated tick bites [49], are less likely to be infected by tick-borne pathogens [53]. Ideally, an anti-tick vaccine would protect against infestation by a wide range of tick species and prevent transmission of multiple tick-borne pathogens. Discussing all tick antigens that have been assessed in vaccination trials is beyond the scope of this article. For an overview of the current stage of development of anti-tick vaccines, there is an excellent review available [54]. An interesting example of an anti-tick vaccine that also protects against the transmission of a tick-borne pathogen is a vaccine targeting the salivary cement protein, 64P, from the tick Rhipicephalus appendiculatus [55,56]. Tick feeding on animals immunized with truncated recombinant forms of 64P (64TRP) resulted in local inflammatory responses and protection against infestation by a wide range of tick species [56]. Importantly, 64TRP-vaccinated mice challenged with tick-borne encephalitis virus (the most important human vector-borne viral infection in Europe [57]) through tick bite were protected from lethal encephalitis [55].

**Figure 2:**
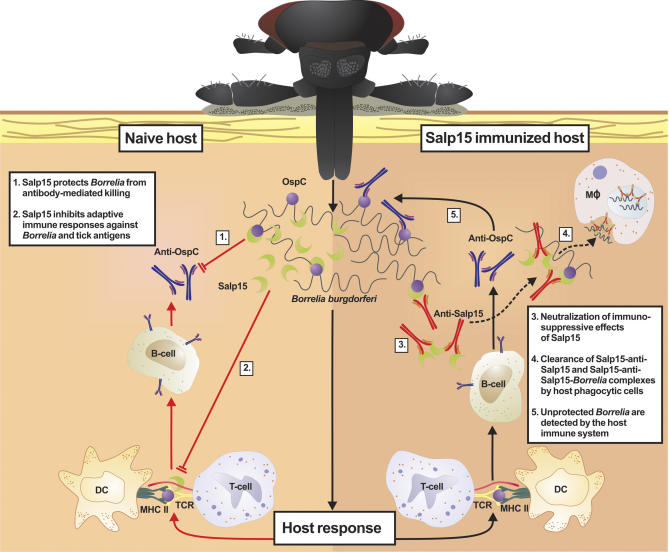
Diagram Showing How an Anti-Salp15 Vaccine Could Prevent Transmission of B. burgdorferi During tick feeding and early mammalian infection, B. burgdorferi expresses OspC, which binds to Salp15 in tick saliva. This binding acts as a shield and protects the spirochete from killing by the host. In addition, Salp15 has been shown to directly inhibit dendritic cell and T cell activation, which could facilitate tick feeding. Salp15 antibodies are likely to bind to Salp15 that has previously bound to OspC on the surface of B. burgdorferi in the tick salivary gland and could thereby enhance clearance by host phagocytic immune cells. Obviously, the Salp15 antibodies would need to recognize a Salp15 epitope other than the epitope that is required for binding of Salp15 to OspC. Similarly, if anti-Salp15 antibodies were to bind to free Salp15, they could neutralize the immunosuppressive effects of Salp15, which could hamper tick feeding and thereby transmission of B. burgdorferi from the tick to the host. Lastly, if anti-Salp15 antibodies were to inhibit binding of Salp15 to Borrelia OspC, this would render the spirochete susceptible to pre-existing or newly generated immunoglobulins. Importantly, Salp15 was originally identified by screening of a tick salivary gland cDNA expression library with tick immune rabbit sera, suggesting that antibodies against Salp15 may participate in tick rejection. DC, dendritic cell; MHC, major histocompatibility complex; MF, macrophage; TCR, T cell receptor.

Proteins that enhance tick feeding may also modulate host immune responses to pathogens, thus playing a double role in transmission. For example, an I. scapularis tick can introduce both Salp15 and B. burgdorferi into the host skin. As described earlier, Salp15 may enhance tick feeding by inhibiting host immune responses to tick antigens. In addition, the B. burgdorferi outer surface protein C (OspC) has been shown to bind to Salp15 in tick saliva [5]. This binding acts as a shield that protects the spirochete against the host immune response (Figure 2). Salp15 would therefore be a candidate to consider for immunization studies. Also, the pleiotropic protein Iris, that not only modulates T cell responses, but also specifically disrupts coagulation [52], could be an interesting candidate. Recently, it was shown that vaccinating rabbits with Iris partially protected these rabbits from tick infestations [58].

## Conclusion

Tick saliva is a potential source for novel pharmacological agents that could be useful for clinical practice. Future research must confirm whether these specific and potent molecules, with promising results in animal models and in human ex vivo experiments, are effective in humans in vivo. The molecules discussed are only a selection of the many physiologically active molecules that have been identified and characterized. However, this selection illustrates the impressive resourcefulness that ticks display to modulate host processes, and demonstrates how we could use these molecules to our benefit. Undoubtedly, future research on tick–host and tick–host–pathogen interactions will reveal even more potential molecules that could be used in clinical practice.

## Abbreviations

64TRP: truncated recombinant forms of 64P
BIP: B cell inhibitory protein
C3: complement factor 3
C5: complement factor 5
FVIIa: activated factor VII
FXa: activated factor X
OspC: outer surface protein C
rTAP: recombinant forms of TAP
TAP: tick anticoagulant peptide
TF: tissue factor
TFPI: TF pathway inhibitor
